# Split GFP technologies to structurally characterize and quantify functional biomolecular interactions of FTD-related proteins

**DOI:** 10.1038/s41598-017-14459-w

**Published:** 2017-10-25

**Authors:** Chiara Foglieni, Stéphanie Papin, Agnese Salvadè, Tariq Afroz, Sandra Pinton, Giona Pedrioli, Giorgio Ulrich, Magdalini Polymenidou, Paolo Paganetti

**Affiliations:** 1 Laboratory for Biomedical Neurosciences, Neurocenter of Southern Switzerland, Torricella-Taverne, Switzerland; 2 Institute of Molecular Life Sciences, University of Zürich, Zürich, Switzerland

## Abstract

Protein multimerization in physiological and pathological conditions constitutes an intrinsic trait of proteins related to neurodegeneration. Recent evidence shows that TDP-43, a RNA-binding protein associated with frontotemporal dementia and amyotrophic lateral sclerosis, exists in a physiological and functional nuclear oligomeric form, whose destabilization may represent a prerequisite for misfolding, toxicity and subsequent pathological deposition. Here we show the parallel implementation of two split GFP technologies, the GFP bimolecular and trimolecular fluorescence complementation (biFC and triFC) in the context of TDP-43 self-assembly. These techniques coupled to a variety of assays based on orthogonal readouts allowed us to define the structural determinants of TDP-43 oligomerization in a qualitative and quantitative manner. We highlight the versatility of the GFP biFC and triFC technologies for studying the localization and mechanisms of protein multimerization in the context of neurodegeneration.

## Introduction

Protein mutations in Mendelian forms of neurodegenerative disorders, aberrant post-translational modifications and pathogenic conformations, all contribute to the progressive accumulation of protein inclusions. These protein assemblies initiate a chain of adverse events ultimately leading to neuronal dysfunction, synaptic loss, cell death, and brain function deterioration. A prion-like process, i.e. accumulative protein deposition, proteotoxicity and transcellular spreading of pathogenic protein forms is typical of most neurodegenerative disorders including Alzheimer’s (AD), Parkinson’s (PD), Huntington’s disease (HD), frontotemporal dementia (FTD) and amyotrophic lateral sclerosis (ALS)^1,2^. The molecular events protecting against proteotoxicity into adulthood or, subsequently, steering proteotoxicity during disease are only in part understood. For example, soluble oligomeric intermediates, rather than deposited amyloid fibrils, may represent the toxic protein forms^3–5^. However, the identification and classification of toxic oligomers is challenging. Some proteins associated with neurodegeneration present a physiological multimeric conformation (e.g. SOD1^6^, α-synuclein^7,8^, TDP-43^9^), and their dissociation may cause a loss of function or may represent a prerequisite for assembly into toxic species. To understand the molecular mechanisms driving neurodegeneration, it is crucial to investigate proteins with regards to how, when and where they (self-)interact to accomplish specific functions or to build the first assemblies into toxic species.

We explored the use of fluorescence reconstitution for live tracking of protein-protein interactions as a tool for elucidating the molecular mechanisms involved in the formation of protein assemblies. Fluorescent sensors are applied to determine protein interactions in cells. One prominent example is FRET from donor to acceptor fluorophores coupled to binding partners^10,11^. Another example is complementation of polypeptide fragments that restore enzymatic activity or fluorescence when in close proximity^12,13^, e.g. the reconstitution of green fluorescent protein (GFP). The bimolecular GFP fluorescence complementation (biFC) requires association of two non-fluorescent fragments followed by reconstitution of the fluorophore^14^. Because assembly of the two fragments in the typical β-barrel conformation of GFP is virtually irreversible^15,16^, biFC leads to the formation, accumulation and detection even of weak or transient protein interactions^17^. Furthermore, the use of a large “sensor” fragment (GFP_1–10_) together with a small GFP fragment composed of a single β-strand (the S_11_ protein tag)^18^ reduces the risk of interfering with the biology of the S_11_-tagged protein of interest. This is an advantage of biFC compared to direct fusion with a relatively large fluorescent protein. Moreover, this approach displays high flexibility because the use of the small tag offers a simple technical strategy allowing direct comparison of different proteins or multiple variants of the same protein^19^, specific detection of subcellular protein pools^20^, and reconstitution into fluorescent proteins with different emission properties^21^. A further advancement of this technique was achieved with the newly developed trimolecular fluorescence complementation (triFC) technology^22^. Here, two consecutive single β-strands (T_10_ and T_11_) are used to each tag one of two binding partners. Protein-protein binding orients the two β-strands so that the concomitant presence of the third fragment (the GFP_1–9_ sensor) will reconstitute fluorescence both *in vitro* and in the living cell. Optimization of the amino acid sequence of the various GFP fragments was required both for biFC and triFC so that the sequence of T_11_ has two amino acid substitutions and is slightly longer than that of S_11_^22^. A further advantage is represented by low background fluorescence due to minimal spontaneous reconstitution^22^.

We present the adaptation of these two technologies for the molecular characterization of protein assemblies that play a critical role in neurodegenerative disorders. As model proteins we selected microtubule-associated Tau and TAR-DNA-binding protein 43 (TDP-43), two proteins independently involved in protein misfolding disorders such AD, PD, ALS and FTD^23,24^. At least 50 mutations in the MAPT gene encoding for Tau are associated with hereditary FTDP-17 but not to AD; whereas neurofibrillary tangles made of hyperphosphorylated Tau are characteristic of AD^25^. Most Tau-negative FTD cases show neuronal changes caused by ubiquitinated and phosphorylated TDP-43, a nuclear protein with a role in transcription, RNA stability and splicing^26^. More than 40 mutations of TDP-43 are associated with the FTD/ALS spectrum of disorders^27^. For these two proteins, we first show their subcellular localization and their self-interaction profiles in living cells. Then, we demonstrate that the parallel implementation of biFC and triFC permits extensive characterization of protein complexes due to a choice of orthogonal, independent read-outs. The combination of triFC with immune assays and flow cytometry results in robust quantitative measures for such interactions in cells. Moreover, the flexible exploitation of the T_11_-tag both for triFC and biFC allows for ratiometric normalization of the triFC data with the level of protein expression determined by biFC. Most importantly, we demonstrate the use of triFC for determining not only protein self-assembly, but more specifically to define the binding between protein domains and their interaction determinants at the molecular level.

## Results

### GFP biFC efficiently localizes proteins in cells

**Figure 1:**
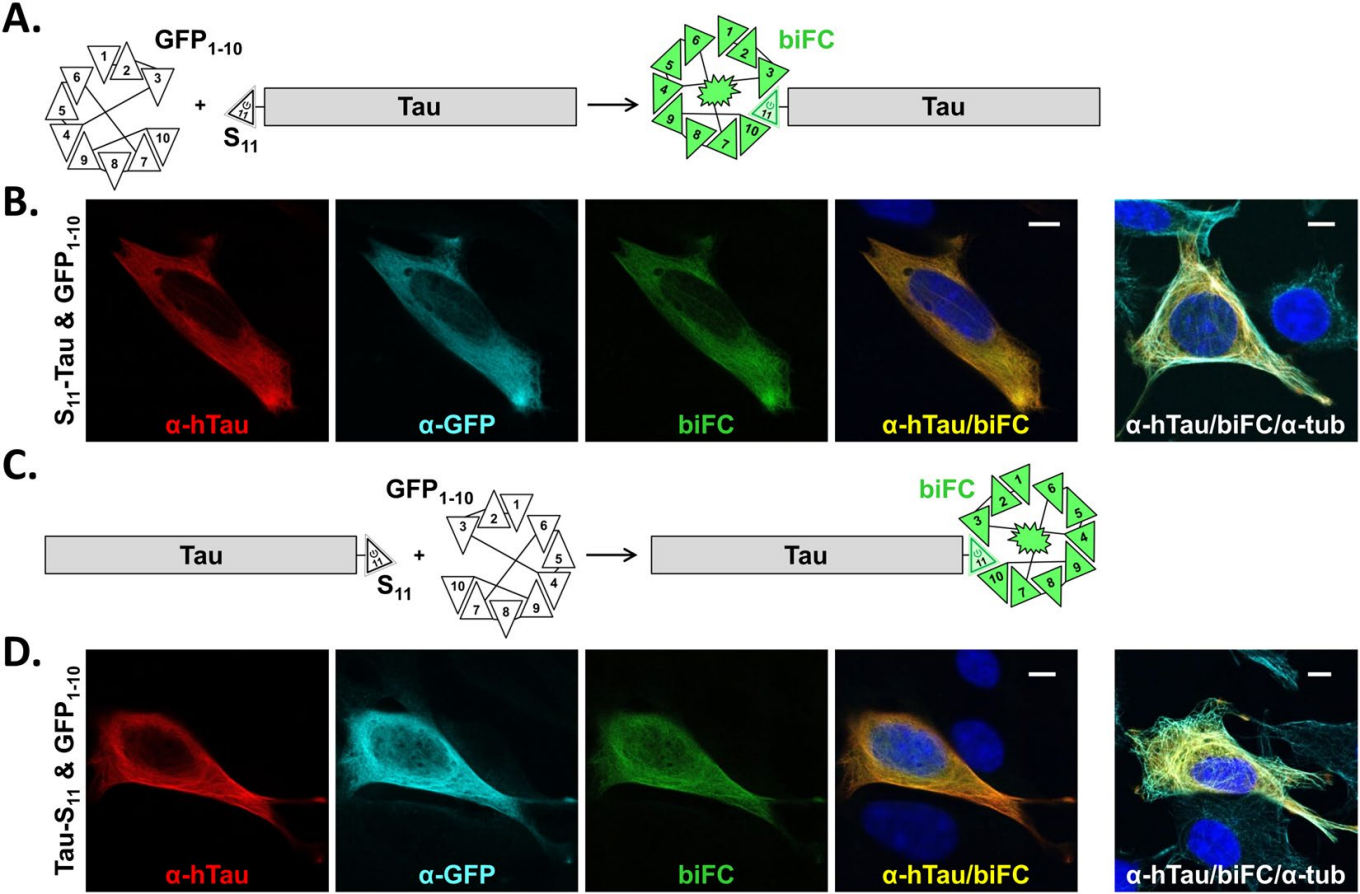
Cellular localization of Tau revealed by GFP biFC. (**A**) Schematic rendition of GFP bimolecular fluorescence complementation (biFC) resulting from co-localization of an N-terminal S_11_-tagged protein (in this case human Tau) with GFP_1–10_. (**B**) Confocal microscope images of methanol-fixed mouse C17.2 cells transiently transfected with S_11_-Tau and GFP_1–10_. The two proteins were immune stained with a monoclonal antibody against human Tau (α-hTau; red dye) and a rabbit antiserum against GFP (α-GFP; cyan dye). Co-localization of S_11_-Tau with GFP_1–10_ reconstitutes GFP and results in biFC (green biofluorescence). BiFC overlaps the distribution of Tau (α-hTau/biFC merge; nuclei counter-stained with DAPI in blue). Also, biFC and α-hTau-staining are found along microtubules, visualized with an antiserum against α-tubulin (α-tub; cyan dye; image on the far right). (**C** and **D**) Same as above but for C-terminally tagged Tau-S_11_. Scale bars: 10 µm.

The GFP biFC technology used herein requires splitting an optimized GFP into two fragments of substantial different size, the 216 amino acid-long fragment encompassing the first ten β-strands of GFP (GFP_1–10_) and the 16 amino acid-long eleventh β-strand of GFP (S_11_), which in turn is used as a relatively small protein tag^18^. The 441 amino acid-long isoform of human Tau was thus tagged with S_11_ at the N- (S_11_-Tau) or C- (Tau-S_11_) terminus (Fig. 1). For both tagged variants, the S_11_ β-strand of GFP was spaced from Tau by a relatively short, nine amino acid-long linker (see Supplementary Fig. S1). S_11_-Tau and Tau-S_11_ were first expressed separately by plasmid transfection in C17.2 cells, a mouse multipotent neural progenitor cell line. One day later, human Tau expression was analysed by immune staining with the human-specific TAU13 antibody^28,29^, thereby detecting the transfected human protein, but not endogenous mouse Tau. In the absence of the GFP_1–10_ sensor, Tau immune staining showed a characteristic cytoskeleton-like distribution in the transfected cells, as expected for this microtubule binding protein (see Supplementary Fig. S1). Human Tau staining was absent in the surrounding, not transfected, cells positive for the nuclear DAPI staining. These data confirmed that the presence of the small S_11_ tag at one or the other Tau end, the heterologous use of human Tau in mouse cells and the CMV promotor-driven expression, did not cause an overt redistribution of Tau in C17.2 cells.

S_11_-Tau was then co-expressed with the GFP_1–10_ sensor (Fig. 1A) and C17.2 cells were stained with the TAU13 antibody (Fig. 1B; dye emission in red) in combination with the α-GFP antibody (Fig. 1B; dye emission in cyan), demonstrating co-expression of the two proteins in transfected cells. As expected, co-localization of S_11_-Tau and GFP_1–10_ reconstituted biFC (Fig. 1B; green biofluorescence). The distribution of biFC perfectly matched that of immune stained Tau, as shown by the computed sum of the two confocal images (Fig. 1B; in yellow). A rabbit polyclonal antibody for α-tubulin specifically revealed microtubules and the distribution of biFC and immune stained Tau along these structures (Fig. 1B; photomicrograph on the right). Consistent with these data, a microtubule-like localization of biFC was also observed in living cells imaged by confocal microscopy (see Supplementary Fig. S1). Undistinguishable data were gained when the C-terminally tagged Tau-S_11_ was used instead of the N-terminally tagged S_11_-Tau (Fig. 1C and D). We conclude that biFC offers an elegant and simple solution for demonstrating the presence and visualizing the subcellular location of Tau in living cells using a modular short tag with limited risk of affecting the normal Tau function.

### GFP triFC reveals specific multimeric protein assemblies in cells

As a next step and based on the observations and conclusions made for biFC, we explored the possibility to visualize protein multimerization by triFC. We first prepared parental plasmids each encoding for one of the two C-terminal consecutive β-strands T_10_ and T_11_ of GFP^22^. Each β-strand is followed by a peptide linker engineered to carry a short antibody epitope (see Supplementary Fig. S2), in order to facilitate the independent analysis of the fusion constructs. The cDNAs encoding for T_10_HA or T_11_β1 were inserted upstream of the multiple cloning region of an expression plasmid in order to facilitate one-step in-frame subcloning of cDNAs encoding a protein of interest. The amino acid sequence of the eleventh β-strand of GFP used for the triFC application, namely T_11_, was slightly different from S_11_ for the biFC^22^. Thus, for comparative purposes, we generated also a parental plasmid encoding for S_11_ followed by the same β1-linker as for T_11_ (see Supplementary Fig. S2).

**Figure 2:**
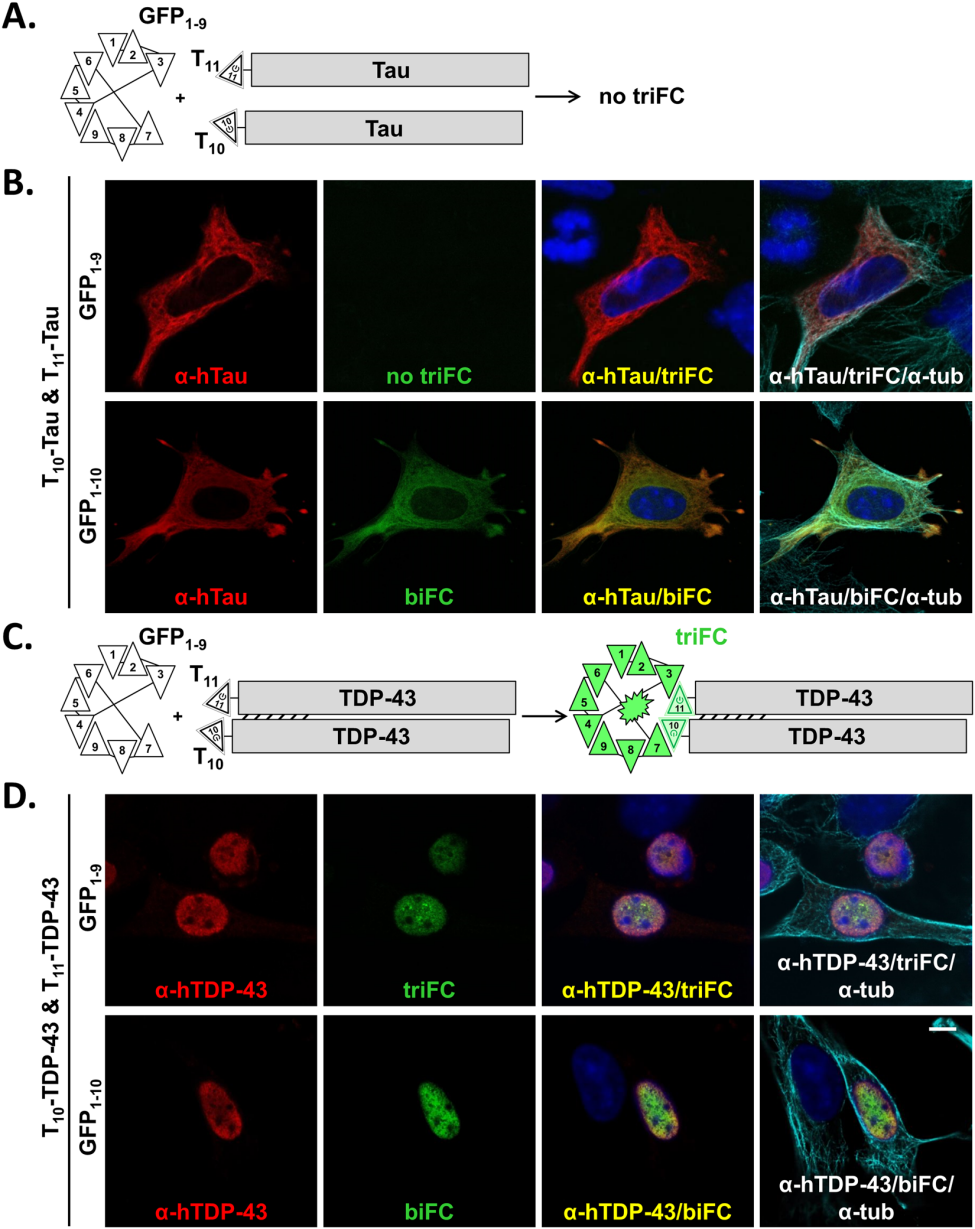
Cellular localization of TDP-43 multimers revealed by GFP triFC. (**A**) Schematic rendition of two variants of human Tau each independently tagged at the N-terminus with T_10_ and T_11_, co-expressed with the GFP_1–9_ sensor but not leading to GFP reconstitution. (**B**) Confocal images of C17.2 cells transfected with T_10_-Tau, T_11_-Tau and GFP_1–9_ (upper row) or GFP_1–10_ (lower row). (**C** and **D**) Same as in (**A** and **B**) but for TDP-43 instead of Tau. Dimerization of the tagged TDP-43 proteins steers T_10_ and T_11_ in an orientation and distance allowing triFC. The presence of Tau or TDP-43 was confirmed by immune staining with the corresponding specific antibody (red dye). In contrast to Tau that does not generate a triFC signal, expression of T_10_-TDP-43, T_11_-TDP-43 and GFP_1–9_ in the same cell results in triFC (green biofluorescence) co-localizing with the α-TDP-43 staining in the nucleus of transfected cells. Microtubule-associated Tau and nuclear TDP-43 reconstitute biFC when tagged with T_11_ and co-transfected with GFP_1–10_. For the merged images on the right, an antiserum to α-tubulin was used (α-tub; cyan dye). Scale bar: 10 µm.

Because of the propensity of Tau to form oligomeric and fibrillar multimers^13,25^, we then generated expression plasmids for the two Tau variants T_10_HA-Tau and T_11_β1-Tau and tested their potential to generate triFC when co-transfected with the optimized GFP_1–9_ sensor^22^ in C17.2 cells (Fig. 2A). One day after transfection, Tau staining confirmed its association to the cytoskeleton, independently visualized by an α-tubulin antibody (Fig. 2B). Co-expression of T_10_HA-Tau, T_11_β1-Tau and GFP_1–9_ in cells resulted in weak, mostly negative, triFC at the confocal microscope (Fig. 2B; top row). These data indicated that under our experimental conditions, detection of self-assembly of N-terminal tagged Tau was not a frequent event in C17.2 cells. In contrast, co-expression of T_10_HA-Tau and T_11_β1-Tau with the GFP_1–10_ sensor resulted in strong biFC for the T_11_β1-Tau/GFP_1–10_ (Fig. 2B; bottom row), just like the S_11_-Tau/GFP_1–10_ complex (Fig. 1B). This showed that the T_11_ β-strand reconstituted green fluorescence in complex with GFP_1–10_ as efficiently as the S_11_ β-strand and indicated accessibility of this Tau region for protein interactions. The failure to observe triFC was attributed to the inability of the two GFP strands fused at the N-terminus of Tau to come in proximity. This could indeed reflect lack of Tau self-assembly under our experimental conditions, a distant organization of the N-termini in the quaternary structure of Tau, or steric hindrance preventing reconstitution. Therefore, we decided to test another FTD-linked protein forming ordered, functional oligomers inside cells^9^.

Despite distinct biological functions, TDP-43 and Tau are implicated in two clinically related inherited variants of FTD, FTLD-TDP-43 and FTDP-17T, respectively^23,24^. T_10_HA-TDP-43 and T_11_β1-TDP-43 plasmids were tested for triFC when co-transfected with GFP_1–9_ in C17.2 cells (Fig. 2C). Transfected cells displayed the typical nuclear distribution of human TDP-43 by immune staining with a human-specific antibody for TDP-43 (Fig. 2D; top row, dye emission in red). Remarkably, in contrast to Tau, a considerable number of transfected cells displayed strong triFC for N-terminal tagged TDP-43 (Fig. 2D; top row, biofluorescence in green). Immune stained human TDP-43 and triFC for TDP-43 localized within the nucleus (Fig. 2D; top row, merged image), consistent with primarily nuclear localization of TDP-43^30^. Expression of the two TDP-43 tagged variants with GFP_1–10_ also resulted in a nuclear signal produced by the T_11_β1-TDP-43/GFP_1–10_ biFC complex (Fig. 2D; bottom row, biofluorescence in green). As expected, the absence of either T_10_HA-TDP-43 or T_11_β1-TDP-43 prevented the formation of the triFC-positive ternary complex (see Supplementary Fig. S2). The amino acid sequence of the S_11_ β-strand of GFP did not substitute that of the T_11_ β-strand for triFC when expressed in C17.2 cells in the presence of T_10_ and GFP_1–9_ (see Supplementary Fig. S2). On the contrary, both T_11_β1-TDP-43 as well as S_11_β1-TDP-43 produced a strong nuclear biFC signal in the presence of GFP_1–10_ (see Supplementary Fig. S2). The data demonstrated that the triFC signal derived from the trimolecular T_10_HA-TDP-43/T_11_β1-TDP-43/GFP_1–9_ complex was highly selective and reflected the subcellular localization and N-terminal domain (NTD)-mediated formation of multimeric nuclear TDP-43 assemblies^9^. In contrast, the biFC signal faithfully reflected the subcellular distribution of either Tau or TDP-43 when tagged with the eleventh β-strand of GFP, independently of the S_11_ or T_11_ sequence.

To rule out artifactual GFP_1–9_ driven interactions, we produced recombinant GFP_1–9_ (see Supplementary Fig. S3). Indeed, triFC occurred when adding recombinant GFP_1–9_ post-fixation to permeabilized HEK-293 and C17.2 cells expressing T_10_HA-TDP-43 and T_11_β1-TDP-43 (see Supplementary Fig. S3 and data not shown). The presence of both TDP-43 forms in the same cells was confirmed by immune staining with the α-HA rabbit antiserum and the mouse β1 monoclonal antibody specific for the epitopes inserted in the respective linkers. No post-fixation triFC was obtained for cells expressing only T_11_β1-TDP-43. These data indicated that self-assembly of TDP-43 in the cell nucleus occurs physiologically in the absence of co-transfected GFP_1–9_.

### Biochemical validation of cellular protein-protein interactions

**Figure 3:**
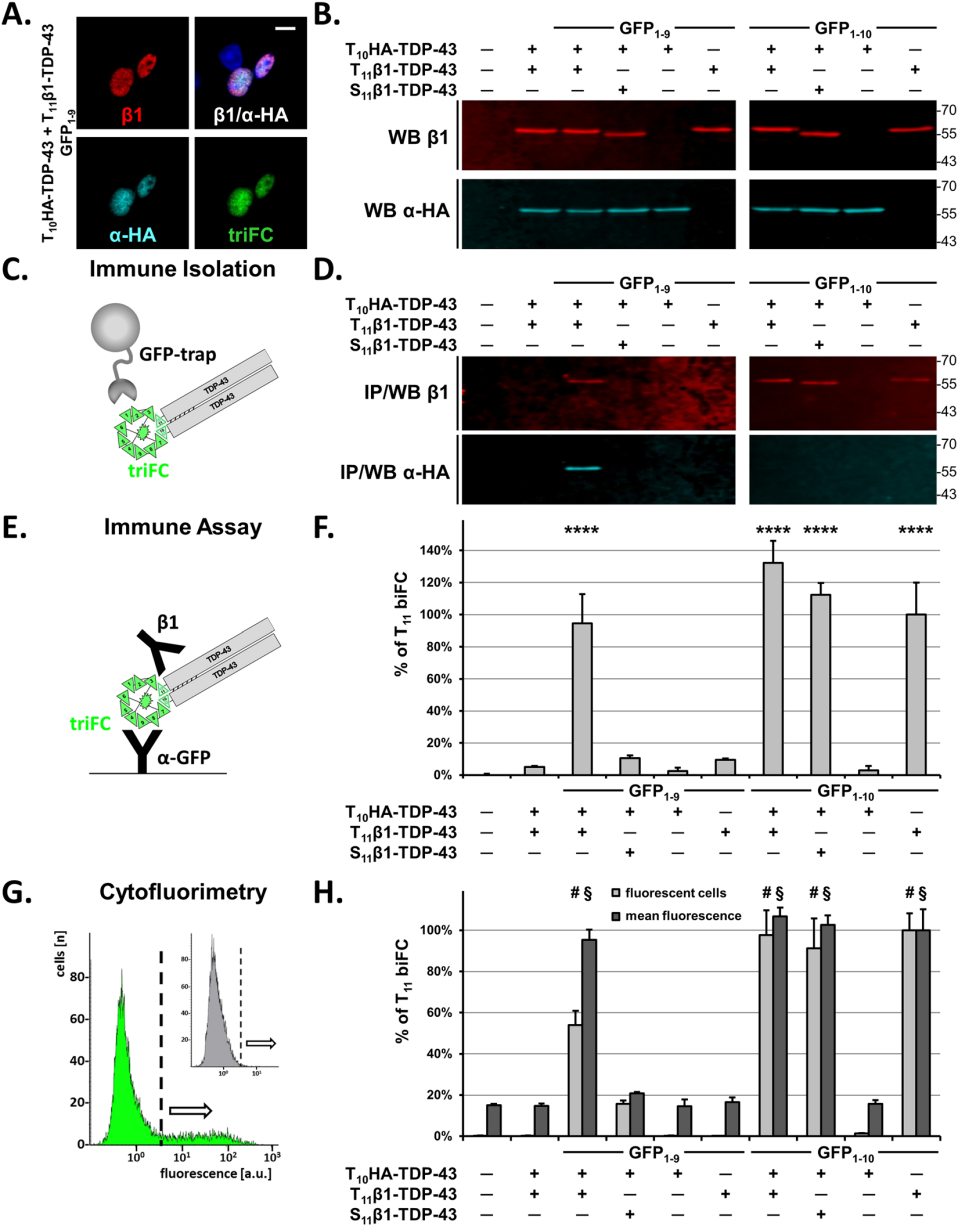
Biochemical and quantitative analysis of the GFP triFC complex. (**A**) Confocal images of HEK-293 cells transfected with T_10_HA-TDP-43, T_11_β1-TDP-43 and GFP_1–9_ plasmids and immune stained with the β1 mouse antibody (red dye) and the α-HA antiserum (cyan dye). Their co-localization (β1/α-HA merge) reconstitutes the triFC ternary complex (green biofluorescence). Scale bar: 10 µm. (**B**) T_10_HA-TDP-43 and T_11_β1-TDP-43 (just above the 55 kDa marker) were also detected in RIPA lysates of cells transfected with the indicated plasmids by duplex-western blot with the β1 (upper panels, red) and α-HA (lower panels, cyan) antibodies. (**C**) Scheme of the immune isolation procedure for the triFC complex using the GFP trap system. (**D**) Western blot analysis of the immune isolates obtained from the cell lysates prepared for (**B**), showed the isolation of the triFC complex containing T_11_β1-TDP-43 (β1 blot) and T_10_HA-TDP-43 (α-HA blot) but not for the negative controls, or when replacing T_11_ with S_11_ (blot on the left). T_11_β1-TDP-43 and S_11_β1-TDP-43 were isolated in the presence of GFP_1–10_ (blot on the right). (**E**) Scheme of the biFC/triFC solid-phase immune assay with an α-GFP antiserum as capture and β1 as detection antibody. (**F**) The data obtained with the α-GFP/β1 immune assay for the cell lysates analysed in (**B**) were consistent with the immune isolation data shown in (**D**). Background value (0%) was determined for mock transfected cells, whereas the value obtained for the GFP_1–10_/T_11_β1-TDP-43 dimer was defined as 100% (% of T_11_ biFC). (**G**) Before lysis, 10,000 transfected cells were analysed by cytofluorimetry. The plots show mean fluorescence versus cell number for mock-transfected cells (inset) or cells expressing the T_10_HA-TDP-43, T_11_β1-TDP-43 and GFP_1–9_ triFC complex. The threshold value for fluorescent cells (dotted vertical line) was arbitrarily defined at the fluorescence value corresponding to 0.05% false positive hits for mock transfected cells. (**H**) Percent fluorescent cells (light grey) and mean GFP-fluorescence (dark grey) obtained by cytofluorimetry of cells expressing the indicated proteins. The value of T_11_β1-TDP-43/GFP_1–10_ cells was defined as 100% for both read-outs. (**F** and **H**) Percent values are means with standard deviations of biological triplicates and one-way ANOVA followed by Dunnett’s multiple comparisons to mock cells. Adjusted P values ****; ^#^; ^§^ < 0.0001.

We demonstrated the selective spatial reconstitution of a triFC-positive T_10_HA-TDP-43/T_11_β1-TDP-43/GFP_1–9_ complex in both mouse C17.2 and human HEK-293 cells. Because of the generally higher expression obtained by plasmid transfection in HEK-293 cells, we chose this cell system for the optimization and implementation of quantitative assays for biFC and triFC. Cells were transfected with T_10_HA-TDP-43, T_11_β1-TDP-43 and GFP_1–9_ or specificity controls including empty plasmid mock transfection, absence of the sensor GFP_1–9_, only one of the two tagged (T_10_ or T_11_) TDP-43 binding partners, or S_11_β1-TDP-43 instead of T_11_β1-TDP-43 (Fig. 3). The co-expression of GFP_1–9_, T_10_HA-TDP-43 and T_11_β1-TDP-43 resulting in positive triFC signal was first monitored by confocal microscopy (Fig. 3A). For all conditions, the presence or absence of both tagged TDP-43 proteins was confirmed in RIPA lysates by duplex-western blotting using the β1 and α-HA antibodies in pooled cell lysates from biological triplicates (Fig. 3B). Cell lysates were then processed with a GFP-specific cameloid single-chain antibody bound to magnetic beads (Fig. 3C). The immune-isolated complex was washed and the α-GFP bound material was eluted by boiling in the presence of SDS. The immune-isolated samples were then analysed by denaturing SDS-PAGE by β1 and α-HA duplex-western blot. The immune precipitation and western blot (IP/WB) procedure led to the detection of the GFP trimolecular complex containing T_10_HA-TDP-43 and T_11_β1-TDP-43 (Fig. 3D). None of the tagged TDP-43 forms was present in the immune isolates obtained from the specificity controls (Fig. 3D). In contrast, when GFP_1–10_ was present instead of GFP_1–9_, T_11_β1-TDP-43 and S_11_β1-TDP-43, but not T_10_HA-TDP-43, were recovered in the immune isolates (Fig. 3D). These data showed that the presence or absence of a triFC signal observed by fluorescence microscopy faithfully reflected the extent of trimolecular GFP complex reconstitution evaluated biochemically.

### Quantification of cellular protein-protein interactions using bi/triFC

Prompted by the biochemical results, we undertook the development of analytical procedures for a quantitative assessment of TDP-43 self-assembly based on triFC. For this, we developed sandwich immune assays for determining reconstituted GFP complexes or their components utilizing antibody pairs against different members of the triFC complex. For the first assay, a rabbit antiserum specific for GFP was adsorbed to the microtiter plates in order to capture GFP_1–9_ or GFP_1–10_. The β1 monoclonal antibody was then used to determine the amount of T_11_β1- or S_11_β1-TDP-43 bound to one or the other GFP sensors (Fig. 3E). With this assay, we processed each individual cell lysate from the biological triplicates analysed as pooled samples by IP/WB. Consistent with the IP/WB data (Fig. 3D), in the presence of the GFP_1–9_ sensor the only significant signal for the GFP/β1 complex was obtained for cells co-transfected with T_10_HA-TDP-43 and T_11_β1-TDP-43, but not for the negative controls (Fig. 3F; P = 0.0001). In contrast, reconstitution of the biFC complex was observed for all cells expressing GFP_1–10_ in combination with T_11_β1- or S_11_β1-TDP-43 (Fig. 3F). Replacing the β1 detection antibody with a monoclonal antibody specific for human TDP-43 produced virtually the same outcome (see Supplementary Fig. S4). The exception was a marginally significant value (P = 0.014) for the T_10_HA-TDP-43/S_11_β1-TDP-43/GFP_1–9_ complex, confirming the strongly reduced - but detectable - penchant of S_11_ to generate triFC when compared to T_11_. These data also showed that T_10_, T_11_, or S_11_ did not bind to GFP_1–9_ in the absence of the matching β-strand of GFP. The presence of the β1 tagged TDP-43 in all samples was confirmed with a TDP-43/β1 assay for all cells transfected with T_11_β1- or S_11_β1-TDP-43, independently of the presence of GFP_1–9_ or GFP_1–10_ (see Supplementary Fig. S4).

To corroborate the quantitative immune assay data with another independent quantitative analysis, we used cytofluorimetry. This technique allowed determining the relative number of triFC-positive cells and their mean fluorescence intensity within a large sample of living cells. The gate for triFC (or biFC) positive cells was defined based on the mock-transfected conditions as maximal five false positive counts among the 10,000 counts analysed (Fig. 3G). All values were then calculated as percent ± standard deviation of the value obtained for biFC cells transfected with T_11_β1-TDP-43 and GFP_1–10_ (100 ± 8% positive cells; 100 ± 10% mean fluorescence intensity corresponding to a mean fluorescence of 4.71 ± 0.48 a.u., i.e. 46-fold higher than the mean fluorescence measured for the negative cells below the gate with a mean fluorescence of 0.10 ± 0.01 a.u.) without subtracting the background values obtained for mock-transfected cells (0.2 ± 0.1% false positive cells; 15 ± 1% mean fluorescence intensity corresponding to a mean fluorescence of 0.71 ± 0.03 a.u.). Expression of T_10_HA-TDP-43, T_11_β1-TDP-43 and GFP_1–9_ resulted in 54 ± 7% of triFC positive cells, which displayed 95 ± 5% fluorescence intensity (both readouts P = 0.0001). None of the other conditions, in the presence or absence of GFP_1–9_, resulted in substantial differences from mock-transfected cells (Fig. 3H). This was also the case for cells transfected with the T_10_HA/S_11_β1-TDP-43 pair, confirming that S_11_ cannot effectively replace T_11_ in complementing T_10_ and GFP_1–9_ in the triFC complex. Consistent with the previous results, close to maximal values were obtained for biFC in cells transfected with GFP_1–10_ in combination with T_11_β1-TDP-43 or S_11_β1-TDP-43 (Fig. 3H). Thus, we inferred that the biFC signal could represent an adequate read-out for determining the expression level of proteins tagged with T_11_ and thus for normalizing triFC when performing comparative studies. We conclude that quantification using triFC and biFC is an accurate method for measuring in parallel protein expression and protein-protein interactions in living cells.

### Quaternary structural information of protein assemblies from triFC

**Figure 4:**
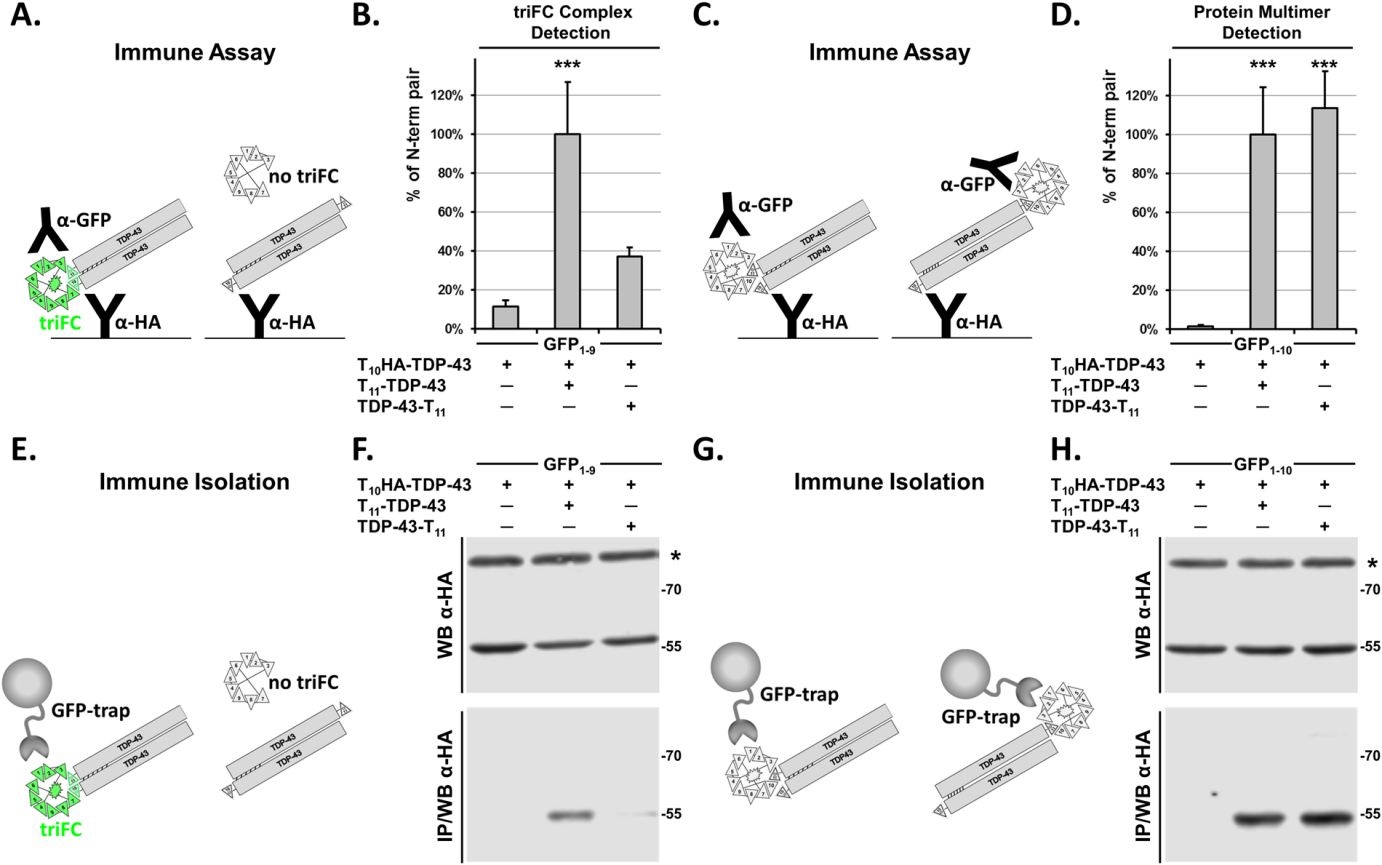
C-terminal T_11_ blocks triFC but not TDP-43 multimerization. (**A**) Scheme of the solid-phase immune assay detecting the ternary triFC complex in the presence of GFP_1–9_ captured with an α-HA antibody and detected with an α-GFP antiserum. (**B**) Cell lysates analysed with the α-HA/α-GFP immune assay show that T_10_HA-TDP-43 forms the triFC complex in the presence of T_11_-TDP-43 but not of TDP-43-T_11_, or in its absence. (**C**) Scheme of the solid-phase immune assay for protein multimers in the presence of GFP_1–10_. T_10_HA-TDP-43 is captured with an α-HA antibody and associated T_11_-β1-TDP-43 bound to GFP_1–10_ is detected with an α-GFP antibody. (**D**) T_10_HA-TDP-43 binds equally well to T_11_-TDP-43 and TDP-43-T_11_. For (**B** and **D**) cells were lysed under mild detergent conditions in order to preserve self-assembled TDP-43. (**B** and **D**) show mean values with standard deviations of biological triplicates with the N-terminally tagged TDP-43 pair defined as 100%. One-way ANOVA and Dunnett’s multiple comparisons to the negative control. Adjusted P values *** < 0.001. Same conditions were also analysed by western blot with an α-HA rabbit antiserum before (WB) or after (IP/WB) immune isolation with the GFP trap system in the presence of GFP_1–9_ for the triFC complex (**E** and **F**) or GFP_1–10_ for protein multimers (**G** and **H**). The protein labelled with an asterisk in (**F** and **H**). results from an unspecific reaction of the α-HA antibody in cell lysates (WB) that it is not immune isolated by the GFP trap system (IP/WB).

Collectively, our data indicate the efficacy of triFC in conjunction with independent (orthogonal) read-outs not only for visualizing specific TDP-43 assemblies inside cells, but also for the quantitative determination of intermolecular interactions. We anticipated that triFC might additionally provide quantitative information on how different domains of a modular protein, such as TDP-43, contribute to self-assembly. To assess this, we analysed the effect on triFC and multimerization when switching the T_11_ tag from the N-terminus to the C-terminus of TDP-43 with an α-HA/α-GFP immune assay or by IP/WB (Fig. 4). We recently reported that triFC of TDP-43 is strongly affected by the location of T_10_/T_11_ tags, which is in line with our high resolution structure of TDP-43 oligomers that form via asymmetric head-to-tail inter-molecular interactions^9^.

HEK-293 cells were thus transiently transfected with T_11_-TDP-43 or TDP-43-T_11_ in the presence of T_10_HA-TDP-43 and GFP_1–9_ whereas the absence of T_11_-tagged TDP-43 served as negative control. One day later, cells were lysed and analysed with the α-HA/α-GFP immune assay. The experiment was run as biological triplicates and the average value for the triplicates were given as percent of the N-terminal pair ± standard deviation (100.0 ± 26.9%), which was significantly different (P = 0.0009) from the negative control obtained in the absence of T_11_-tagged TDP-43 (11.5 ± 3.0%) (Fig. 4B), consistent with the above-described immune assays (Fig. 3F and see Supplementary Fig. S4). More interestingly, we found that co-expression of the N-terminally tagged T_10_-TDP-43 with the C-terminally tagged TDP-43-T_11_ reconstituted poorly (37.1 ± 4.7%), the triFC complex - without reaching statistical significance (P = 0.16) - in the presence of GFP_1–9_ (Fig. 4B). In contrast, in the presence of GFP_1–10_ that together with T_11_ reconstitutes biFC, co-transfection of the N-terminal pair (100.0 ± 24.2%; P = 0.0009) or the N-terminal T_10_/C-terminal T_11_ pair (113.5 ± 18.9%; P = 0.0005) resulted in similarly positive significant values (Fig. 4D), illustrating the accessibility of the T_11_ tags in both cases. In line with this, IP/WB as immune isolation of the triFC complex was much more efficient for the N-terminal pair when compared to the N-terminal T_10_/C-terminal T_11_ pair (Fig. 4E and F). In contrast, the α-GFP coated beads isolated efficiently T_10_-TDP-43 associated with both the N-terminally and C-terminally T_11_-tagged TDP-43 bound to GFP_1–10_ (Fig. 4G and H). Altogether, these data verified our hypothesis that triFC was specific for determining TDP-43 self-assembly only when the two complementary β-strands are in proximity and with the correct orientation to permit GFP reconstitution. This occurred when T_10_ and T_11_ were fused at the N-terminus of TDP-43, a result consistent with the involvement of the NTD of TDP-43 in mediating oligomerization^9^. Instead, the α-HA/α-GFP immune assay performed on lysates obtained from cells expressing GFP_1–10_, detected TDP-43 self-assembly independently of the orientation of the two β-strands of GFP. This occurred because T_10_HA-TDP-43 was captured by the α-HA-specific antibody, whereas the presence of the co-isolated T_11_-tagged TDP-43 was revealed by the GFP_1–10_/α-GFP detection system. Altogether, our triFC data combined with orthogonal readouts show the versatility of the method to characterize quaternary structural organization of modular-domains containing protein such as TDP-43.

### Validation of inter-molecular interface within protein assemblies with triFC

**Figure 5:**
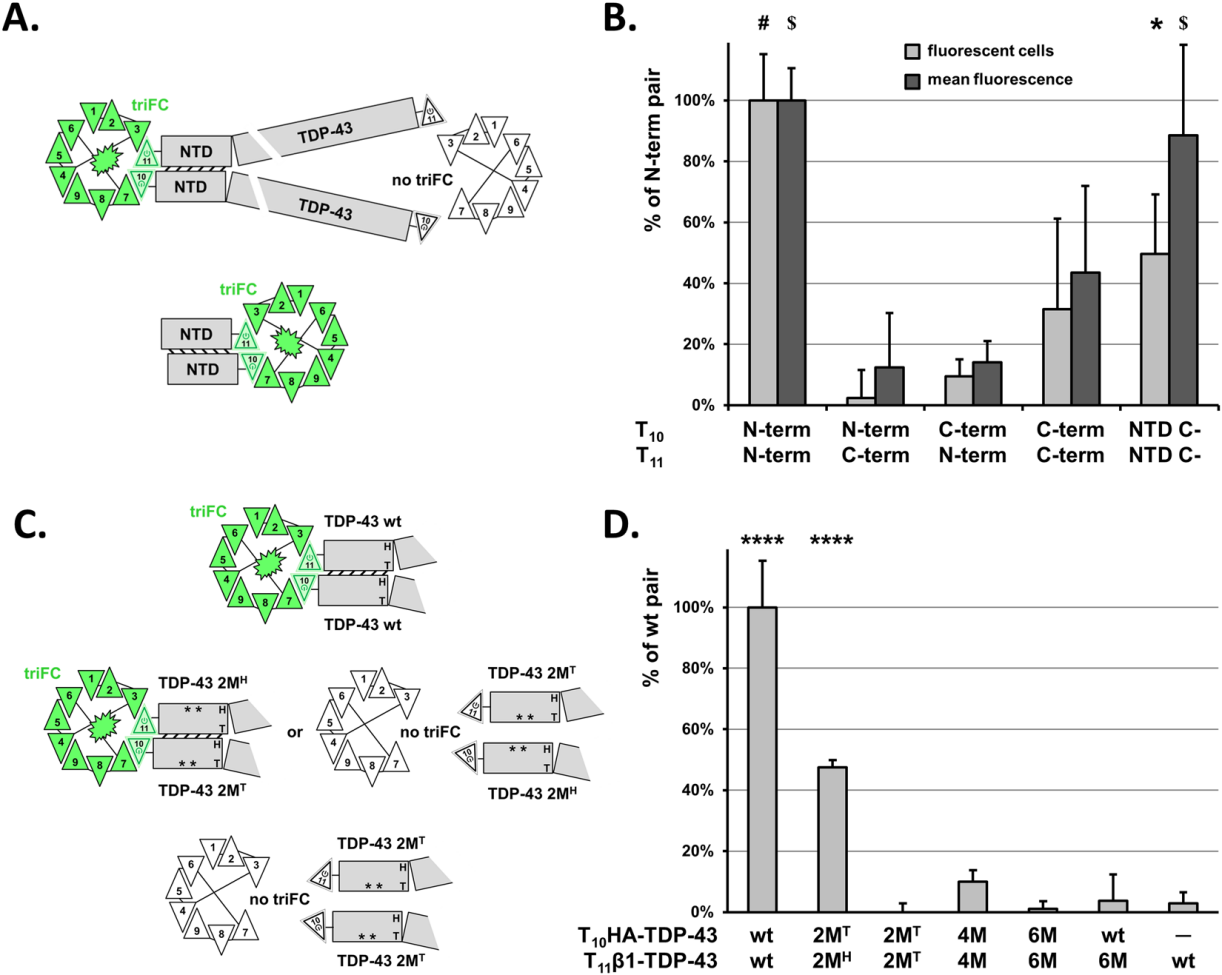
TDP-43 multimerization is driven by its N-terminal domain. (**A**) Schematic representation for the selective detection of TDP-43 interacting domains by GFP triFC. (**B**) Cytofluorimetric analysis of relative fluorescent (triFC-positive) cells (light grey) and their mean GFP-fluorescence (dark grey) for 10,000 human SH-SY5Y neuroblastoma cells transfected with the indicated plasmids. Only the N-terminal tagged TDP-43 pair and the C-terminal tagged NTD pair reconstitute triFC. Values are means with standard deviations of biological triplicates shown as percent of the N-terminal TDP-43 pair. All triFC values were normalized with the respective biFC values obtained in the presence of GFP_1–10_ as measure of T_11_-tagged TDP-43 expression. One-way ANOVA followed by Dunnett’s multiple comparisons to mock transfected cells. Adjusted P values * < 0.05; # < 0.0001; $ < 0.001. (**C**) Scheme representing the perturbation of TDP-43 multimerization (compared to the wt pair; top) caused by the insertion of point mutations within the NTD and expected to partly (2 M^H^/2 M^T^ pair; middle) or completely (2 M^T^/2 M^T^ pair; bottom) impair TDP-43 self-assembly. (**D**) SH-SY5Y cells were transfected with the indicated combinations of mutant T_10_HA-TDP-43 or T_11_β1-TDP-43 in the presence of GFP_1–9_. Cell lysates were then analysed with the α-GFP/β1 solid-phase immune assay for the triFC complex and normalized for T_11_β1-TDP-43 determined with the α-TDP-43/β1 immune assay. Values are means with standard deviations of biological triplicates and are expressed as percent of the value obtained for the wt pair. One-way ANOVA and Dunnett’s multiple comparisons to the negative control. Adjusted P values **** < 0.0001.

To further assess the biological relevance of these results, we tested all possible combinations of N- and C-terminal T_10_- or T_11_-tagging of TDP-43 for reconstitution of triFC in SH-SY5Y cells, a human cell line of neuronal origin. Cells were thus transiently transfected with the four combinations of tagged TDP-43 and 24 h later they were analysed by cytofluorimetry for reconstituted GFP fluorescence (Fig. 5A and B). The values of each TDP-43 pair in the presence of the GFP_1–9_ sensor (triFC) was normalized for the values obtained in the presence of the GFP_1–10_ sensor (biFC) as a surrogate measure of T_11_-tagged TDP-43 expression. The average values for three independent experiments were reported as percent of the N-terminally tagged pair. This was also the only TDP-43 pair for which statistically significant values in terms of number of fluorescent cells (p = 0.0001) and mean fluorescence (P = 0.0002) were obtained when compared to the N-/C-terminal mixed pairs (Fig. 5B). These data obtained in SH-SY5Y cells were consistent with the qualitative data reported for mouse C17.2 cells^9^, and were coherent with therein described physiological self-assembly of TDP-43.

Nuclear TDP-43 oligomers represent the functional form of the protein and are assembled by the stacking of contiguous NTDs^9^. Notably, this active form of TDP-43 appeared to position the respective C-terminal regions sufficiently distant from each other^9^ so that no substantial interaction was detected by triFC for the C-terminal pair (Fig. 5B). Much in contrast, a significant amount of fluorescent cells (50 ± 20%, P = 0.012) displaying a significant mean fluorescence (89 ± 30%, P = 0.0004) were recovered for the C-terminal pair when TDP-43 was truncated at the end of the NTD (Fig. 5B). Overall, these data showed that the combination of triFC/biFC read-outs could illustrate which protein domains interact in living cells in a quantitative manner.

The data also support a model where the NTD is sufficient to initiate and drive the self-assembly of TDP-43 in the absence of the RNA binding domains. In order to better analyse this process, we assessed the use of the methodologies we developed for the identification of the molecular determinants of NTD self-assembly based on our recently published structural data^9^ of TDP-43 in oligomeric state. These data showed the molecular interactions of two contiguous NTDs of TDP-43 resulting in a head-to-tail mode of oligomer assembly, and in particular specified the critical role of two amino acids in the head and four amino acids in the tail. We thus tested specific mutations in the head (2 M^H^; R52A and R55A), in the tail (2 M^T^; E17A and E21A), both combined (4 M) or with six mutations (6 M; E14A, E17A, E21A, Q34A, R52A and R55A) of N-terminally tagged T_10_/T_11_-TDP-43 (Fig. 5C and D) for their effect in reconstituting the triFC complex in human SH-SY5Y cells using the α-GFP/β1 immune assay. The GFP/β1 signals were normalized for T_11_β1-TDP-43 expression determined with the α-TDP-43/β1 immune assay. Finally, the values obtained for the mutant pairs were calculated as percent of wild-type TDP-43 (wt; 100 ± 15%, P = 0.0001). Consistent with the structural data, mutation on both T_10_- and T_11_-tagged TDP-43 molecules of two (2 M^T^/2 M^T^; −1 ± 45%), four (4 M/4 M; 10 ± 4%), or six (6 M/6 M; 1 ± 3%) of the amino acids involved in NTD interaction interface was sufficient to completely impair TDP-43 self-assembly and thus triFC reconstitution. Mutation of six amino acids on only one of the two TDP-43 members of the triFC complex (wt/6 M; 4 ± 9%) also blocked triFC reconstitution (Fig. 5D). Based on the proposed model, two NTDs assemble through two interfaces placed one on the head and one at the tail of the domain. Consistent with the head-to-tail model of NTD self-assembly, combining a TDP-43 pair where one partner carried two mutations on the head and one partner carried two mutations in the tail should limit TDP-43 to dimers (Fig. 5C). Indeed the triFC signal for the 2 M^H^/2 M^T^ pair was significantly rescued (47 ± 2%; P = 0.0001) albeit reduced by about half when compared to the wt pair (Fig. 5D). The difference in triFC complex reconstitution between the blocked dimer (2 M^H^/2 M^T^ pair) and wt TDP-43 indicates that triFC distinguishes these two forms that present different propensity for oligomeric growth.

Altogether these results demonstrate the versatility of bi/triFC in conjunction with orthogonal immune assays and cytofluorimetry as a method to characterize and quantify protein-protein interactions in cells as well as to obtain structural information of protein assemblies inside cells.

## Discussion

Cellular functions entail the matched action of proteins in multimeric assemblies and cellular pathways defined as molecular machines. Depending on the biological process, proteins interact with their binding partners with a wide range of affinities leading to either transient or long-lived complexes. The modification of the quaternary state of a protein complex can be regulated e.g. by ligand binding or more general variations, such as those occurring under stress conditions^31^. In this study, we investigated TDP-43, a FTD- and ALS-associated protein that incorporates in at least three distinct assemblies. Functional nuclear homo-oligomers entail the interaction of adjacent NTDs of TDP-43, bind nucleic acids and catalyse mRNA splicing^9^. Cytosolic membrane-less granules form in response to cellular stress from mRNA and mRNA-binding proteins, including TDP-43, interacting through their intrinsically disordered domains^32^. Finally, during disease progression TDP-43 accumulates in pathological inclusions in dysfunctional neuronal and glial cells^27^. Therefore, the study of protein-protein interactions is crucial for understanding cell function in health and disease.

Many methodologies have been developed to characterize multimeric protein assemblies. These include cell-free approaches such as affinity-based procedures, surface plasmon resonance or calorimetry^33,34^. Genetic screens (two-hybrid systems, phage-display assays) are also used to define interactomes^35,36^. Paradigms to investigate protein interactions in living cells were designed more recently, including BRET^37^, FRET^10,11^, and protein complementation restoring enzymatic activity^12,13^ or biofluorescence^38^. We selected and further developed fluorescence complementation in order to better understand which qualitative and quantitative information, and which limitations this technique may offer when studying native biomolecular interactions within cells. In particular, we focused on the methodologies developed by Waldo and colleagues^18,22^ since splitting GFP into fragments of substantially different size results in the use of relatively small protein tags for modifying the protein of interest, whilst preserving versatile applications. In fact, triFC represents an advantage over biomolecular based FRET or enzyme complementation because of the reduced size of the tags. triFC is also more advantageous than FRET based on proteins labelled with synthetic fluorophores injected in the living cells, because the GFP-derived tags are only slightly larger than the chemical FRET probes but can be easily expressed in cells^39^. triFC offers the opportunity to localize precisely the subcellular site of protein-protein interaction when compared to enzymatic complementation technologies that generate more broadly dispersed products.

We showed that the information delivered by biFC is mostly limited to detecting and assessing protein expression, as well as investigating its intracellular distribution in living cells. biFC has found application also in protein-protein interaction studies when the GFP_1–10_ sensor (or other FP fragments) was used to tag a protein of interest^40,41^. Nevertheless, spontaneous self-assembly of two FP fragments leads directly to the formation of a high affinity biFC complex, hence increasing the likelihood of false positive signals when compared to triFC.

Upon reconstitution, the two GFP fragments are virtually irreversibly bound to each other so that biFC becomes very similar to a simple fusion of the protein of interest to the intact GFP. Post-fixation fluorescence reconstitution may even solve the limitation of irreversible reconstitution, and thus it may be employed as an end-point read-out. Moreover, biFC presents attractive features in terms of flexible choice of where to place the tagging β-strand (at both ends but also within the protein of interest), and the use of the same β-strand for reconstituting fluorescent proteins with different emission wavelengths^21^. Alternatively, biFC represents a powerful technology to detect the appearance of a protein in atypical subcellular locations, such as the appearance of a cytosolic protein in the lumen of an organelle^20,21^. We also provide evidence that biFC is suited for quantitative read-outs that are advantageous when e.g. normalizing for protein expression. Indeed, we demonstrated that S_11_ can be replaced with T_11_ making this latter the sequence of choice both for biFC and triFC. Ultimately, the use of biFC is not limited to transfected cells but it can be adapted to study endogenous proteins^19^.

For triFC, we obtained evidence that fluorescence reconstitution from GFP_1–9_ is not driven only by the interaction of a T_10_-protein with a T_11_-tagged binding partner^22,42,43^. Rather, triFC attests that the two interacting proteins dictate the correct spatial orientation and distance of the two β-strands T_10_ and T_11_. Thus, the choice of spacer length defines the likelihood of ternary fluorescence reconstitution to the point that a sufficiently long spacer would make the position of the β-strands irrelevant when studying protein assembly^22,43^, although this would need to be evaluated against the risk of losing the active contribution of the interacting partners to the reconstitution process. Conversely, the influence of spacer length abbreviation or elongation on the efficiency of triFC delivers information on the position and distance between domains of interacting partners or belonging to the same protein^43^.

The cytofluorimetric data establish that in transiently transfected cells the mean fluorescence intensity generated by triFC is very similar to that generated by biFC, signifying that reconstituted GFP from two or three fragments delivers complemented molecules with similar fluorescent emission efficiency. In contrast, the number of cells positive for triFC was about half as high as that obtained for biFC. Suboptimal positioning of two β-strands, weak or transient protein interaction or the fact that an intramolecular reaction between three components instead of two is less likely to occur may explain this difference. It should be noted that although triFC complex formation is primarily driven by the interaction of the two proteins tagged with the T_10_ or T_11_ β-strands of GFP, the further addition of the GFP_1–9_ sensor leads to an irreversibly assembled complex^22^. In order to standardize protein modification with T_10_ or T_11_, we generated parental plasmids for N- or C-terminal tagging of a protein of interest. Each β-strand and spacer nucleotide coding sequence contained single-restriction sites simplifying the engineering of sequences encoding different epitopes or when modifying the spacer length or composition. The four tagged versions of the same protein can each be singularly detected by antibodies against each of the four epitopes contained in the different tags.

We adapted biFC and triFC for different, orthogonal read-outs, some of which provide quantitative assessments of protein expression and interaction. Multiple read-outs, in particular when the analysis by fluorescence is complemented for the same samples with fluorescence-independent read-outs, reduce substantially the likelihood of false negative and positive outcomes, such as intra- or inter-molecular fluorescence quenching. An additional important aspect was the possibility to quantify triFC in intact cells, followed by complementary biochemical investigations of the triFC complex after cell lysis. In principle this could also serve to analyse protein assembly-specific post-translational modifications or protein conformation assuming the availability of specific antibodies. Quantitative read-outs for triFC complemented with biFC-based normalization for protein expression were particularly informative when studying TDP-43 in cells. In this respect, we expect that the flexibility and robustness offered by triFC is a specific attribute of this technology when compared to alternative technologies to study protein-protein interaction in cells.

Our data confirm that Tau is found mostly associated with the microtubular cytoskeleton. Addition of small sequences at the N- or C-terminus did not overtly interfere with microtubule binding. Using triFC, we were not able to detect the formation of Tau multimeric assemblies, indicating the absence of Tau multimers under our experimental conditions or wrong orientation of the β-strands. It should be noted that in cells expressing high levels of Tau upon transient transfection, we observed few cells positive for triFC (not shown), possibly indicating a stochastic process of (irreversible) GFP reconstitution that may reduce the specificity of triFC assay under these conditions. We showed that post-fixation addition of recombinant GFP_1–9_ reconstitutes GFP fluorescence, thus representing a strategy to verify protein multimerization in cells in the absence of GFP_1–9_ sensor.

With regards to TDP-43, confirming the recently published structural and functional evidence^9^, our data demonstrate the critical role of the NTD of TDP-43 for its functional oligomerization, as exemplified by the nuclear triFC signal observed by confocal microscopy. The importance of the NTD of TDP-43 in assembling an active RNA splicing multimer was shown using a GFP-fusion protein^9^, suggesting a likely functional integrity of the TDP-43 triFC complex. More importantly, we provide experimental evidence in cells for the identification of specific amino-acids in the NTD of TDP-43, which mediate the interface interactions necessary for its self-assembly. This also highlights the ability of biFC/triFC for identifying the interacting domains of proteins and the molecular determinants of this interaction. The fact that triFC requires an accurate tailoring for positioning the complementing GFP chain on the protein of interest is more challenging but represent an unique and informative feature offered by this technology when compared to alternative strategies such as BRET or biomolecular FRET. We expect that the use of the reconstituted GFP as an immune isolation bait may become instrumental for the specific identification of post-translational modifications of interacting partners within a molecular complex.

The triFC technology combined with the orthogonal assays described in this report offer a unique way to study the physiological and pathological states of other proteins implicated in neurodegeneration. Mutations in various RNA binding proteins such as hnRNP A1, hnRNP A2/B1, FET protein family (FUS, TAF-15, EWSR1) Matrin-3, TIA-1 and Ataxin-2^44^ are implicated in various neurodegenerative diseases. Similarly to TDP-43, the modular-domain architecture of these RNA binding proteins not only allows the incorporation of T_10_ and T_11_ tags at the extreme N- or C-termini but also in the flexible regions such as inter-domain linkers, thereby specifically positioning the tags in proximity to the desired domain. Therefore, in addition to investigating direct protein-protein interactions, the technology can further be expanded to analyse indirect interactions such as those depending on a cofactor, such as RNA, for the above-described proteins in neurodegeneration.

Overall the data obtained demonstrate the utility of technologies based on fluorescence reconstitution in the context of studies aimed at investigating the biochemistry and cell biology of proteins involved in neurodegenerative disorders.

## Materials and Methods

### Expression plasmids

The plasmid pcDNA3 was used as backbone mammalian expression vector for all cDNA constructs in this study. The plasmid encoding GFP_1–10_ was kindly provided by Dr. Tito Calì, University of Padova^20^. It covered the amino acid sequence of the first ten β-strands of an optimized form of the superfolder green fluorescent protein as described^18^. The plasmid for GFP_1–9_ was custom synthesized (GenScript) with optimized mammalian codons and amino acid sequence corresponding to the GFP_1–9_ OPT protein^22^ with exception of two additional amino acids (Asp-Ile) inserted after the initial Met creating a restriction site for EcoRV.

The plasmid encoding for S_11_-Tau was obtained by the polymerase chain reaction (PCR) using as template a synthetic Tau cDNA (Promogène/Texcell). The 105 base-long forward primer 5′TTCGGATCCATGCGGGACCACATGGTGCTGCACGAGTACGTGAACGCCGCCGGCATCACAGGCGACGGCGGCAGCGGCGGCGGCAGCGCTGAGCCCCGCCAGGAG encoding the S_11_ β-strand of GFP followed by a nine amino acid linker and the 32 base-long reverse primer 5′TTCACTCGAGTCACAAACCCTGCTTCGCGAGG were used. The PCR fragment was then inserted in the single-cutting restriction sites BamHI/XhoI present in the poly-linker of the expression plasmid and in the primers (underlined sequences). The plasmid for Tau-S_11_ was kindly generated by Dr Tito Calì (University of Padova) with the forward primer 5′GATCAGGATCCATGGCTGAGCCCCGCC and the reverse primer 5′TCTCACTCGAGTCATGTGATGCCGGCGGCGTTCACGTACTCGTGCAGCACCATGTGGTCCCGGCTGCCGCCGCCGCTGCCGCCGTCGCCCAAACCCTGCTTCGCGAGG. All Tau constructs described in this work encoded for the 441 amino acid-long splice variant 2N4R of human Tau. Fusion Tau constructs were designed in a way that they lacked either the initial methionine when adding an N-terminal sequence or the stop codon for the C-terminal modification.

For generating fusion proteins modified with the tenth (T_10_) or eleventh (T_11_) β-strand of GFP fused at the N- or C-terminus of Tau or TDP-43 for trimolecular GFP complementation^22^, we prepared parental plasmids with custom synthesized DNA fragments (GenScript). The plasmid for N-terminal T_10_HA- carried the sequence 5′AAGCTTACCATGGATCTCCCAGACGATCATTACCTGTCCACCCAGACAATCCTGAGCAAAGATCTTAATGGGGTACCAGGTTACCCATACGATGTTCCAGATTACGCTGGACCTAGCGGCGGTGAGGGCTCAGCCGGCGGAGGACCGGTCGGAGGCGGATCC in the HindIII/BamHI sites of pcDNA3. Plasmid T_11_β1- carried 5′AAGCTTACCATGGAGAAGAGGGACCACATGGTGCTGCTGGAGTACGTGACCGCCGCCGGCATCACCGACGCCTCGGGGGTACCAGGTTCAGAGTTCAGGCACGACAGCGGCGGACCCGGGAGCGGCGGTGAGGGCTCAGCCGGCGGAGGACCGGTCGGAGGCGGATCC. Control plasmid for S_11_β1- carried 5′AAGCTTACCATGCGGGACCATATGGTGCTGCACGAGTACGTGAACGCCGCCGGCATCACAGGGGTACCAGGTTCAGAGTTCAGGCACGACAGCGGCGGACCCGGGAGCGGCGGTGAGGGCTCAGCCGGCGGAGGACCGGTCGGAGGCGGATCC. The sequences encoding for the single β-strands of GFP were followed by peptide linkers (see Supplementary Fig. S2) carrying either the commercial α-HA antibody-epitope or the β1 antibody-epitope developed in the laboratory^45^ but also recognized by the commercial monoclonal antibody 6E10 against a human-specific epitope of the β-amyloid peptide. The plasmid for C-terminal T_10_ followed by the C-terminal Aβ40 epitope was obtained by inserting in the XhoI/XbaI sites of pcDNA3 5′CTCGAGGGCGGACCCGGGAGCGGCGGTGAGGGCTCAGCCGGCGGAGGACCGGTCGGAGGCGGAAGCGGGATATCAGGTTCAATGGATCTCCCAGACGATCATTACCTGTCCACCCAGACAATCCTGAGCAAAGATCTTAATCTCATGGTAGGCGGAGTAGTCTAGA. C-terminal T_11_ followed by the Aβ42 epitope had the sequence 5′CTCGAGGGCGGACCCGGGAGCGGCGGTGAGGGCTCAGCCGGCGGAGGACCGGTCGGAGGCGGAAGCGGGATATCAGGTTCAGAGAAGAGGGACCACATGGTGCTGCTGGAGTACGTGACCGCCGCCGGCATCACCGACGCCTCGGGAGGAGTAGTGATCGCGTAGTCTAGA. All plasmids encoded the complete amino acid sequences of the respective human proteins, including the initial methionine. C-terminal tagging for TDP-43-T_10_ and TDP-43-T_11_ was obtained by eliminating the stop codon of TDP-43 and in frame BamHI/Xhol subcloning into the corresponding parental plasmids, respectively. NTD-T_10_ and NTD-T_11_ covered the 105 amino acid N-terminal fragment of TDP-43.

### Cell culturing and plasmid transfections

Mouse multipotent neural progenitor C17.2 cells (07062902, ECACC), human neuroblastoma SH-SY5Y cells (94030304, Sigma-Aldrich) and human embryonic kidney HEK-293 cells (provided by Prof. Maurizio Molinari, IRB, Bellinzona, Switzerland) were cultured in DMEM (61965-059, Gibco) supplemented with 1% non-essential amino acids (NEAA; 11140035, Gibco) and 10% FBS (10270106, Gibco). Cells were grown in an incubator at 37 °C with saturated humidity and 5% CO_2_. Cells were passaged at confluency with a 1:5–1:20 split.

For plasmids transfection, cells were grown on culture plates coated with poly-D-lysine (P6407, Sigma-Aldrich) to 70–80% confluency, usually reached one day after cell plating. C17.2 and SH-SY5Y cells were transfected with Lipofectamine 3000 (L-3000-008, Invitrogen) or jetPRIME (114-15, Polyplus-transfection) according to the manufacturer instructions. As an example, 17 × 10^3^ C17.2 cells/well were plated on a microscope 8-well slide (80826, Ibidi). One day later, cells were supplemented with 200 μL/well fresh medium. The transfection mixture was prepared by mixing solution 1 made with 0.3 μL Lipofectamine 3000 added to 15 μL Optimem (11058021, Gibco) and pre-incubated at room temperature for 5 min and solution 2 made with 0.3 μg total plasmid DNA diluted to 15 μL with Optimem and 0.3 μL P3000 reagent, pre-incubated at room temperature for 5 min. After additional 10 min, the transfection mixture was given slowly to the cells. Transient transfection in HEK-293 cells was usually performed by plasmid precipitation with calcium phosphate. For this, two equal volumes of solution A and solution B were combined by adding drop-wise solution B into solution A while gently mixing. Solution A (2xHBS) contained 280 mM NaCl, 1.5 mM Na_2_HPO_4_ and 50 mM HEPES. Solution B was prepared by diluting the DNA into 0.25 mM CaCl_2_. Multiple 2xHBS solutions with pH values between 6.96 and 7.04 were prepared in order to select the one that produced a light hazy solution after 10 min incubation at room temperature, i.e. the appearance of fine calcium phosphate crystals when viewed through the microscope. The transfection mixture was then slowly added to the cell medium. The culture medium was replaced 4 h after transfection with fresh medium and the cells incubated for at least one day before further analysis. Cells were usually analysed in live by confocal microscopy prior to fixation and immune staining.

### Immune staining

For immune staining or in live analysis, transfected cells were grown on poly-D-lysine coated microscope 8-well slides. One day after plasmid transfection, cells were fixed in methanol. For this, the culture medium was removed before adding 200 μL/well −20 °C methanol. After 10 min in the −20 °C freezer, the methanol solution was removed by aspiration and the fixed cell layer was first gently washed three times with PBS (10010-056, GIBCO), then blocked with 300 μL/well 5% normal goat serum, 0.3% Triton X-100 in PBS for 30 min and washed again with PBS. All further steps were performed at room temperature with a working solution composed of 0.5% normal goat serum, 0.3% Triton X-100 in PBS. Primary antibodies, usually incubated for 1 h at 37 °C, were specific for human TDP-43 (1 μg/mL; 60019-2-Ig, Proteintech), human Tau (TAU13; 0.66 μg/mL, sc-21796, Santa Cruz), GFP (2.5 μg/mL; ab290, Abcam), α-tubulin (0.5 μg/mL; ab1825, Abcam), α-HA (8 μg/mL; 51064-2-AP, Proteintech) and β1 (2.3 μg/mL). Secondary antibodies were α-mouse IgG (Alexa594; 2 μg/mL; A-11032, ThermoFisher), or α-rabbit IgG (Alexa647; 2 μg/mL; A-21245, ThermoFisher). Nuclei were counter-stained with 0.5 μg/mL DAPI (D9542, Sigma-Aldrich). Incubations were performed for 1 h at room temperature in the dark. Slides were finally washed three times with PBS and stained cells kept in PBS with 0.05% sodium azide in the fridge. Immune stained cells were analysed with a confocal microscope (Confocal Microscope, C2 Nikon). Confocal images were taken with a line by line scan using a sequence of excitation with the 405 nm laser line and emission filter 464/40–700/100 nm (represented in blue), followed by 488 nm laser and 525/50 nm filter (represented in green) and by 561 nm laser and 561/LP nm filter (represented in red). A second scan was performed with excitation with the 640 nm laser and emission filter 464/40–700/100 nm (represented in cyan), which was then digitally combined with the first scan.

### Post-fixation triFC with purified recombinant GFP_1–9_

Cells co-expressing T_10_HA-TDP-43 and T_11_β1-TDP-43 were fixed for 10 min at 37 °C directly in cell culture medium by adding one volume of 4% paraformaldehyde dissolved in PBS adjusted at pH 7.4 with NaOH (PFA/PBS). This was followed by an additional fixation for 5 min at room temperature with 200 μL/well 4% PFA/PBS. After three washes with 100 mM glycin in PBS and one wash with PBS, blocking was performed with 300 μL/well 5% normal goat serum, 0.3% Triton X-100 in PBS for 30 min followed by three PBS washes. Recombinant GFP_1–9_ was diluted at 1 mg/mL in PBS and incubated 4 h at room temperature on the fixed cells. After three PBS washes, cells were immune stained and analysed by fluorescent microscopy (Inverted Research Microscope ECLIPSE Ti-E, Nikon).

For recombinant protein expression in bacteria, the DNA sequence coding for GFP_1–9_ was PCR amplified and cloned into the expression vector - pET-28a(+) with an in-frame N-terminal 6xHis coding sequence. For protein expression, the plasmid was transformed into Escherichia coli BL21 codon plus RIL strain (230240, Agilent). A kanamycin-resistant single colony was inoculated into 50 mL of Luria-Bertani medium (LB) and grown overnight and then diluted into 1 L of LB. The cells were grown at 37 °C to a 0.6–0.8 optical density at 600 nm. The temperature was reduced to 25 °C and the bacterial cultures were induced for 24 h with 1 mM isopropyl β-D-thiogalactoside. Bacterial cultures were collected by centrifugation at 4200 g for 10 min. The cell pellets were resuspended in 20 mL lysis buffer (150 mM NaCl, 100 mM Tris-HCl pH 7.4, 10% (v/v) glycerol, 1 mM DTT, 10 mM imidazole, protease inhibitors) and lysed by sonication on ice with a 0.5 inch diameter probe (Q500, Qsonica sonicator) with 15 sec ON, 45 sec OFF pulses (total sonication ON time 10 min). The lysate was centrifuged at 45,000 g for 50 min. The resulting supernatant containing soluble protein was used for GFP_1–9_ purification under native conditions on ÄKTA Prime purification system (Amersham Biosciences) using Ni^2+^-affinity chromatography (5 mL His-Trap column; GE Healthcare). The column was equilibrated with binding buffer (150 mM NaCl, 100 mM Tris-HCl pH 7.4, 10% glycerol, 1 mM DTT). Following loading of the sample and extensive washing with binding buffer, recombinant GFP_1–9_ was eluted with a linear imidazole gradient in the elution buffer (500 mM imidazole in binding buffer). The recombinant GFP_1–9_ fractions controlled for purity by SDS PAGE were pooled, dialyzed in binding buffer, concentrated at 3200 g with a Vivaspin 10,000 MWCO (Sartorius Stedium Biotech), repurified on a second Ni^2+^-affinity chromatography and stored at −20 °C.

### Cytofluorimetric analysis

For cytofluorimetric analysis, plasmid transfections were performed in 6-well culture plates. Cells were collected by a trypsin treatment and, cautiously resuspended in culture medium in order to obtain a single-cell suspension. Cells were then washed with ice-cold PBS, resuspended in 0.5 mL ice-cold PBS and kept on ice until analysis. Cytofluorimetry for GFP was performed for 10,000 cells on an analytical device (Beckman Coulter, Navios^TM^ Flow Cytometer) using the 488 nm excitation laser and the FL1 emission channel (525/40 nm). Values collected included total cell number, gated cell number and geometric mean fluorescence.

### Immune assays

For quantitation or western blot analysis of the triFC complex, the rest of the cell suspension analysed by cytofluorimetry was centrifuged at 4 °C, 2 min, 1200 rpm with a table-top centrifuge (5417 R, Eppendorf). The cell pellet was lysed in ice-cold 80 µL RIPA buffer (R0278, Sigma-Aldrich) with protease (S8820, Sigma-Aldrich) and phosphatase (04906845001, Sigma-Aldrich) inhibitors for 30 min on a cooling shaker (5355, Eppendorf) at 4 °C and 1400 rpm. The lysates were then incubated on the shaker for additional 15 min at 37 °C after adding 10 units of DNAse (4536282001, Sigma-Aldrich) and MgCl_2_ to 5 mM final concentration. The reaction was stopped with 9 µL 0.25 M EDTA followed by a centrifugation at 4 °C, 10 min, 20,000 g generating a clear cell extract. Protein concentration was determined (23227, Pierce BCA Protein Assay Kit) and adjusted to 1 µg/µL.

For the immune assays, microtiter 96-well plates (M9410, Sigma-Aldrich) were coated overnight in the fridge with 50 µL/well capture antibodies diluted in PBS; α-GFP (5 μg/mL) or α-TDP-43 (0.6 μg/mL). Plates were then washed three times with 200 µL/well 0.05% Tween 20 in PBS (PBS-T), blocked for 1 h at 37 °C with 1% bovine serum albumin (A4503, Sigma-Aldrich) in PBS-T, and washed again four times. Cell extracts were diluted 1:10 with PBS and 50 µL/well added for 1 h at 37 °C, followed by four washes. Detection antibodies β1 (10 μg/mL) or α-TDP-43 (2.1 μg/mL) in PBS-T were incubated for 1 h at 37 °C followed by four PBS-T washes. The immune assay was developed after 30 min incubation with 1:5000 goat α-mouse IgG-HRP conjugate (170–6516, BioRad), four washes, and addition of 3,3′,5,5′-tetramethylbenzidine (T0440, Sigma-Aldrich). The reaction was stopped with 2 M phosphoric acid and the optical density red at 450 nm (iMark, BioRad).

### Immune isolation on beads, immune blotting

Immune isolation was performed using magnetic beads. For this 120 μg total cell extract obtained by pooling biological triplicate samples were brought to a volume of 250 μL with ice-cold RIPA buffer. For each samples 3 μL of 30% slurry GFP-trap beads (gtma-20, ChromoTek) equilibrated in PBS were added and rotated for 2 h at 4 °C. Using a magnet, the beads were then washed once with a 1:1 mixture of RIPA and TBS buffer (150 mM NaCl, 50 mM Tris/HCl) and once with TBS buffer alone. Immune isolates were then collected in 30 μL SDS PAGE sample buffer and incubated at 95 °C for 10 min before protein separation on SDS PAGE.

Cell extracts (15 μg protein) and immune isolates were resolved by 10% SDS PAGE and transferred to PVDF membranes (162-0177, BioRad). Blots were blocked in Odyssey Blocking Buffer (927–50000, LI-COR), and developed for the infrared western blot technology using an Odissey CLx device (LI-COR). Primary antibodies, incubated for 1 h at 37 °C, were α-HA (0.4 μg/mL) and β1 (4.6 μg/mL). Secondary antibodies were α-rabbit IgG coupled to IRDye 800CW (LI-COR) and α-mouse IgG coupled to IRDye 680RD (LI-COR), incubated on membranes for 30 min at 37 °C.

For dimers analysis, cells were collected directly in gentle lysis buffer composed of 0.2% Nonidet-P40, 5 mM EDTA, 10% glycerol with protease and phosphatase inhibitors in PBS^46^ and incubated on ice for 10 min. The lysates were processed with DNAse and after centrifugation the cell extract was collected.

For the immune assays, the same procedure as above was followed, using for capturing an antibody α-HA (0.4 μg/mL) and an α-GFP (5 μg/mL) as detection antibody.

In order to enrich for the protein multimer, 600 μg lysates were incubated with 7 μL of 30% slurry GFP-trap beads. After a 2 h incubation at 4 °C and magnetic separation of beads, immune isolates were collected in 15 μL SDS PAGE sample buffer and incubated at 95 °C for 10 min before SDS PAGE.

Cell extracts (10 μg protein) and the total immune isolates were analyzed by western blot as described, except for the incubation of the α-HA (0.08 μg/mL) overnight at 4 °C and that of the secondary α-rabbit IgG-IRDye 800CW for 1 h at room temperature in the dark.

## Electronic supplementary material

Supplementary Figures
