# Supplementary material for: Split GFP technologies to structurally characterize and quantify functional biomolecular interactions of FTD-related proteins
Source: Sci Rep. 2017 Oct 25;7:14013. doi: 10.1038/s41598-017-14459-w (PMC5656600; doi:10.1038/s41598-017-14459-w)
Supplement: Supplementary file 1 — Supplementary Figures [file 41598_2017_14459_MOESM1_ESM.doc]

**Split GFP technologies to structurally characterize and quantify functional biomolecular interactions of FTD-related proteins**

Chiara Foglieni, Stéphanie Papin, Agnese Salvadè, Tariq Afroz^£^, Sandra Pinton, Giona Pedrioli, Giorgio Ulrich, Magdalini Polymenidou^£^ and Paolo Paganetti*

Laboratory for Biomedical Neurosciences, Neurocenter of Southern Switzerland

^£^ Institute of Molecular Life Sciences, University of Zürich

* Correspondence to:

*Dr. Paolo Paganetti*

*Leader Neurodegeneration Research Group*

*Neurocenter of Southern Switzerland / EOC*

*c/o SIRM, Via ai Söi 24, CH-6807 Torricella-Taverne*

*Tel +41 (0)91 811 7250*

*paolo.paganetti@eoc.ch*


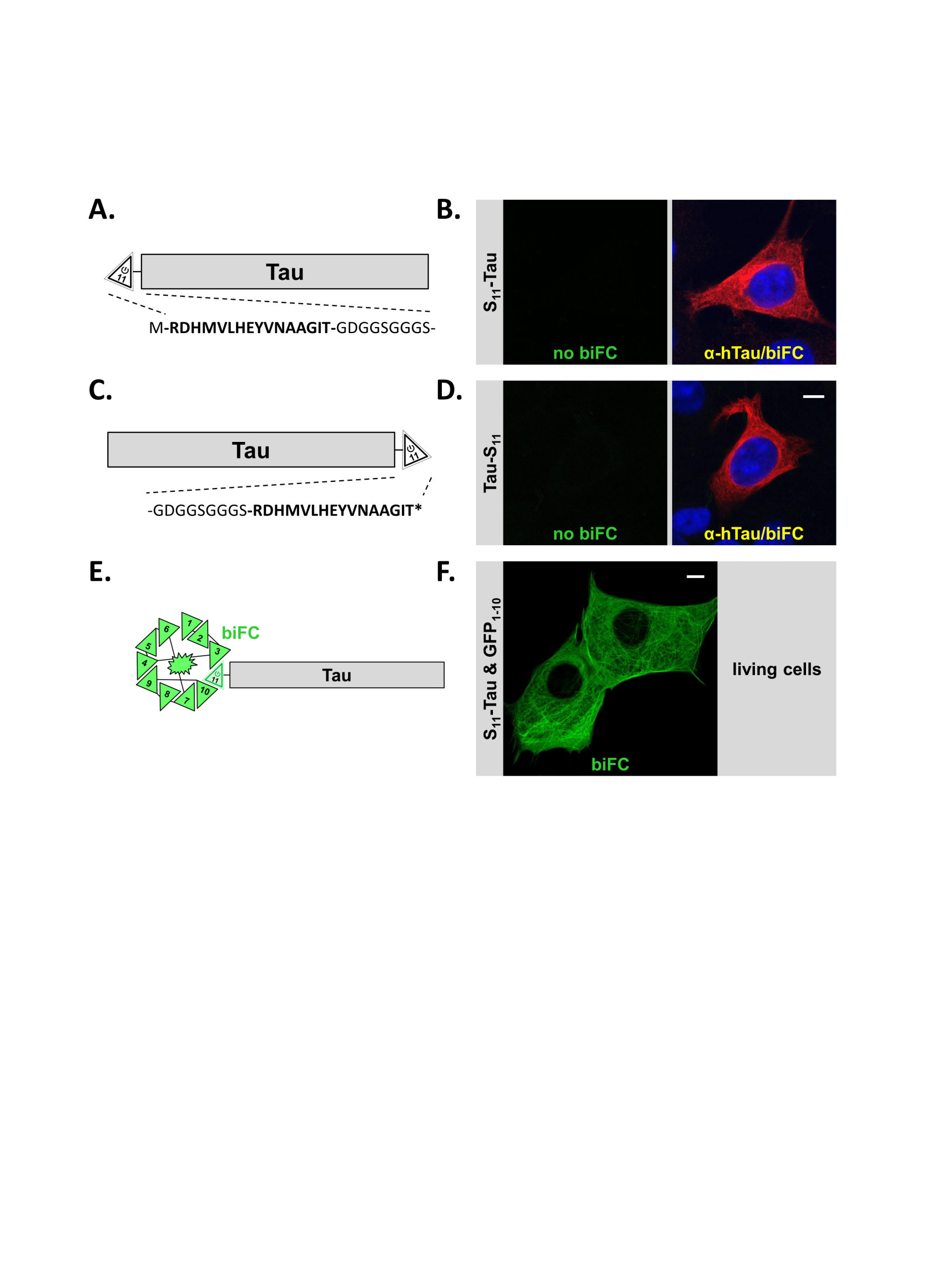


**Suppl. Figure S1. Specificity controls for GFP biFC.**

**A.** Scheme of protein tagging, amino acid sequence of the S_11_ β-strand of GFP (in bold) and the spacer used for N-terminal tagging of a protein of interest for biFC. **B.** Confocal microscope images of mouse neuronal progenitor C17.2 cells transfected with S_11_-Tau, methanol-fixed and immune stained with an α-hTau antibody (red dye, nuclei stained with DAPI in blue); no biFC fluorescence is detected in the absence of GFP_1-10_. **C.** and **D.** Same as in A. and B. but for C-terminal-tagged Tau-S_11_. **E.** Scheme of the S_11_-Tau/GFP_1-10_ biFC complex**. F.** *In live* confocal microscopy of biFC in C17.2 cells forming the S_11_-Tau/GFP_1-10_ complex. Scale bars: 10 µm.

**
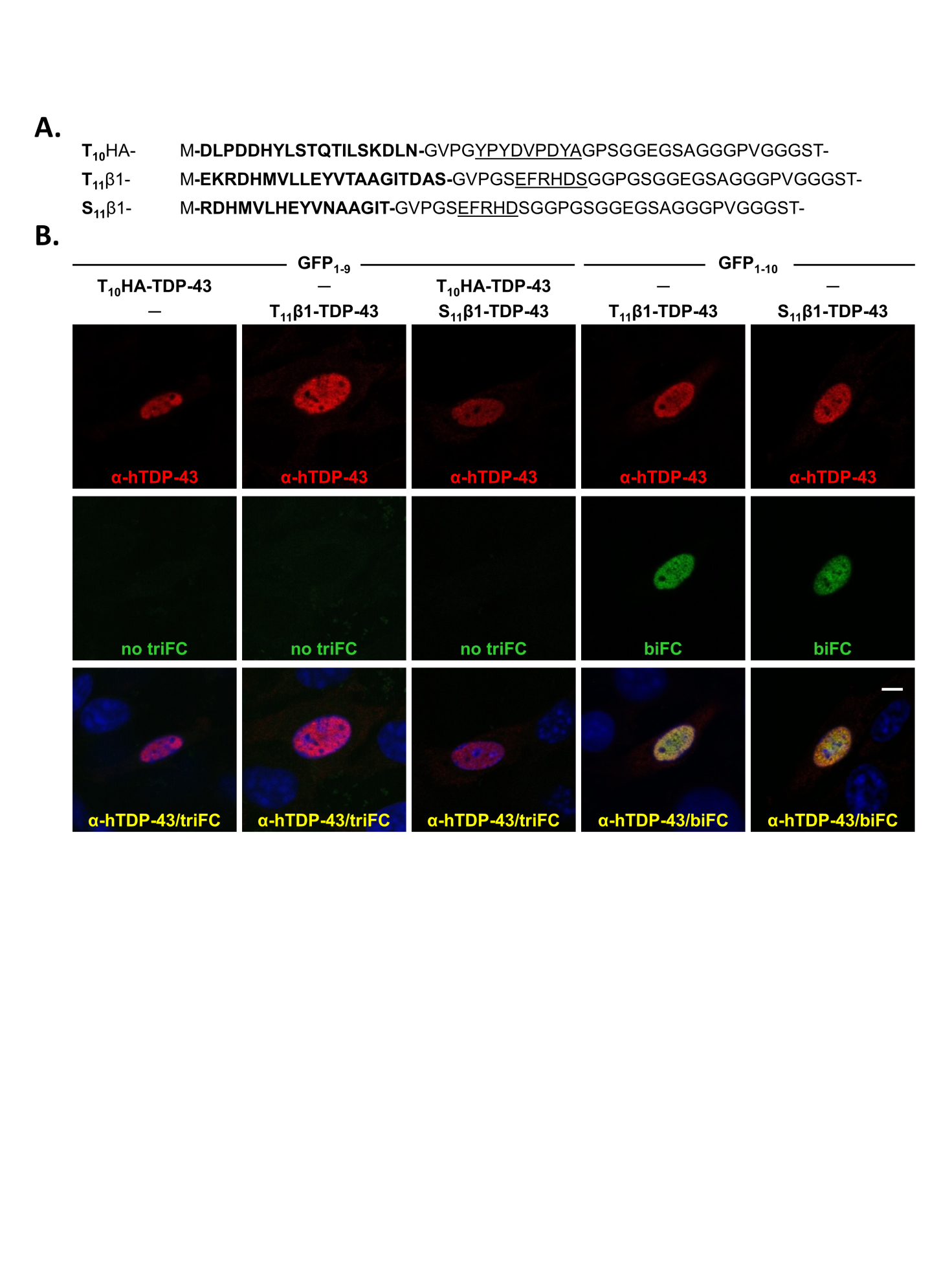
**

**Suppl. Figure S2. Specificity controls for GFP triFC.**

**A.** Amino acid sequences of the T_10_, T_11_ and S_11_ β-strands of GFP (in bold) engineered for the N-terminal tagging of a protein of interest for triFC. The spacers contain sequences recognized by the antibodies α-HA or β1 as indicated (underlined). **B.** Confocal microscope images of C17.2 cells expressing different TDP-43 forms in the presence of GFP_1-9_ or GFP_1-10_ as indicated. The location of human TDP-43 in the nucleus was visualized by immune staining with a human-specific α-TDP-43 antibody (red dye, upper low). No reconstitution of GFP triFC (green channel) was observed in the absence of one of the two biding partners in the ternary complex with GFP_1-9_ or when S_11_β1 was replacing T_11_β1. In contrast, T_11_β1- and S_11_β1- reconstituted GFP in the binary biFC complex in the presence of GFP_1-10_. Merged images display also nuclear staining with DAPI in blue. Scale bar: 10 µm.


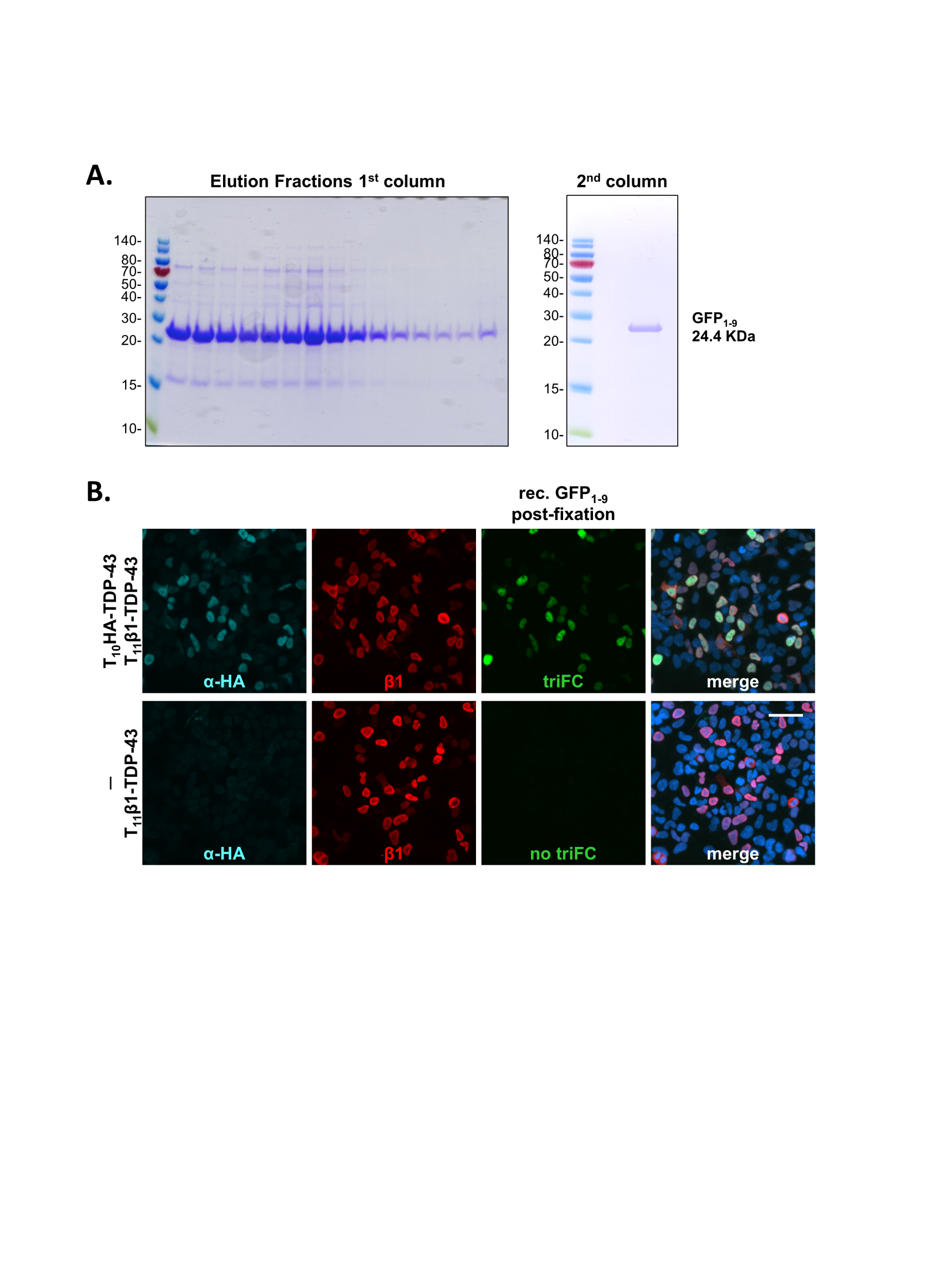


**Suppl. Figure S3. TDP-43 multimers are detected by triFC when recombinant GFP_1-9_ is added to fixed cells.**

**A.** SDS-PAGE analysis of GFP_1-9_ recombinant protein in the elution fractions obtained from the first and second Ni^2+^-affinity chromatography purification. Column elution was performed with a linear imidazole gradient. Recombinant GFP_1-9_ displays the expected ~24KDa apparent molecular weight. **B.** Epifluorescence microscope images of HEK-293 cells transfected with the indicated constructs. One day after transfection cells were fixed and incubated with recombinant GFP_1-9_. Post-fixation reconstitution of GFP triFC occurred in cells transfected with the T_10_HA-TDP-43 and T_11_β1-TDP-43 pair (top row) but not in the absence of T_10_HA -TDP-43 (bottom row) as confirmed by immune staining with α-HA (cyan dye) or β1 (red dye) antibodies. Scale bar: 50 µm.


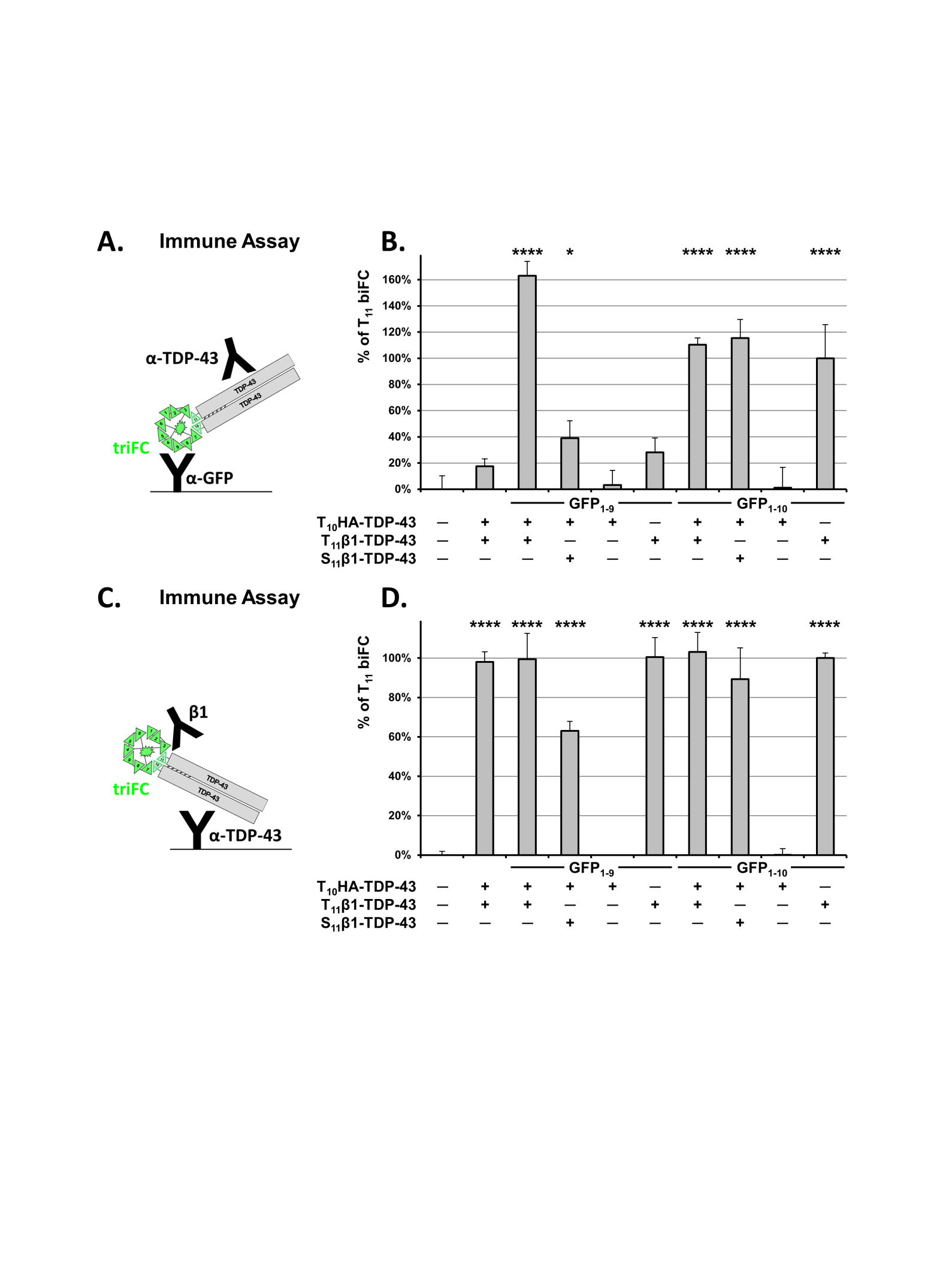


**Suppl. Figure S4. Quantitative immune assays for multimers and total TDP-43.**

**A.** Scheme of the α-GFP/α-TDP-43 immune assay using an α-GFP rabbit antiserum to capture the triFC or biFC complexes and the α-TDP-43 mouse antibody followed by an α-mouse HRP-antibody for detection. **B.** The cell lysates investigated in Fig.3 were analysed with the α-GFP/α-TDP-43 immune assay in order to determine the TDP-43/GFP complex. Background value (0%) was that of empty plasmid mock transfected cells, whereas the value for the T_11_β1-TDP-43/GFP_1-10_ complex was defined as 100%. **C.** Scheme of the α-TDP-43/β1 immune assay for determination of total β1-tagged TDP-43 (in complex with GFP and free). **D.** Assessment of the total amount of T_11_β1-TDP-43 or S_11_β1-TDP-43 in the cell lysates analysed in Fig. 3 and quantified as described for B.. In B. and D**.** show mean values with standard deviations of biological triplicates. Adjusted P values *<0.05; ****<0.0001; one-way ANOVA followed by Dunnett’s multiple comparisons to mock transfected cells.
